# In vitro activity and in vivo efficacy of omadacycline against *Plasmodium* species

**DOI:** 10.1186/s12936-025-05448-w

**Published:** 2025-06-19

**Authors:** Michael S. Madejczyk, Susan E. Leed, Karl Kudyba, Alison Roth, Monica L. Martin, Patricia Lee, Diane Anastasiou, Jessica V. Pierce, Alisa W. Serio, Diana Caridha

**Affiliations:** 1 Experimental Therapeutics Branch, Center of Infectious Disease Research, Walter Reed Army Institute of Research, Silver Spring, MD 20910 USA; 2 ORISE Fellow, Walter Reed Army Institute of Research, Silver Spring, MD 20910 USA; 3 Paratek Pharmaceuticals Inc, King of Prussia, PA 19406 USA

**Keywords:** Omadacycline, Doxycycline, *Plasmodium falciparum*, *Plasmodium cynomolgi*, *Plasmodium berghei*, Antimalarial drugs, Parasitemia, In vivo imaging technology

## Abstract

**Background:**

Doxycycline is currently the only tetracycline-class antibiotic recommended for malaria prophylaxis. Omadacycline, a semisynthetic aminomethylcycline approved for treatment of adults with community-acquired bacterial pneumonia and acute bacterial skin and skin structure infections, has a well-established safety profile. This study evaluated the in vitro activity of omadacycline against *Plasmodium falciparum* and *Plasmodium cynomolgi* and its in vivo efficacy against *Plasmodium berghei* in experimental malaria models to assess its potential as an antimalarial drug.

**Methods:**

Fluorescence-based assays were used to assess the in vitro blood and liver stage activity of omadacycline and doxycycline against *P. falciparum* and *P. cynomolgi* laboratory clones. In vivo liver and early-stage blood stage efficacy were evaluated in a murine model of *P. berghei* infection, utilizing in vivo imaging of luciferase-expressing *P. berghei* (ANKA strain) sporozoites in female albino C57Bl/6 mice. Parasitaemia was monitored by flow cytometry for up to 30 days post-infection.

**Results:**

Omadacycline demonstrated comparable in vitro activity to doxycycline against both drug-sensitive and drug-resistant *P. falciparum* clones, while doxycycline showed reduced activity against two drug-resistant clones. Notably, omadacycline exhibited superior anti-schizont activity in the *P. cynomolgi* liver stage assay. In the *P. berghei* murine model, omadacycline was efficacious in both liver and early blood stages compared to the untreated control group, and demonstrated improved survival compared to doxycycline.

**Conclusions:**

Omadacycline demonstrated enhanced antimalarial efficacy over doxycycline in vitro in liver stage activity and in overcoming resistance in the blood stage, and in survival in an in vivo model of *P. berghei* infection. These findings support further investigation of omadacycline as a potential candidate for malaria prophylaxis and treatment.

## Background

Malaria remains one of the most deadly and widespread infectious diseases globally, with an estimated 263 million cases and 597,000 deaths reported in 2023 [1]. Of the species of parasite responsible for human malaria, *Plasmodium falciparum* is the dominant species in sub-Saharan Africa and is responsible for the majority of malaria-related deaths. *Plasmodium vivax*, responsible for relapsing malaria, is the causative agent of up to 3.5% of the global malaria burden, whereas *Plasmodium ovale* and *Plasmodium malariae* cause a smaller percentage of infections [1]. *Plasmodium knowlesi* primarily infects nonhuman primates but has also led to isolated cases of human malaria. Malaria continues to disproportionately affect children and pregnant women in sub-Saharan Africa, as well as impacting United States (US) and partner nations’ military deployments in both conflict and stability/peacekeeping missions [1, 2]. Recent autochthonous transmission in the US—nine cases reported in 2023—highlights ongoing risks even in non-endemic regions [3]. Additionally, climate change is expected to expand *Anopheles* mosquito habitats, potentially increasing transmission beyond traditional endemic zones [1].

The three most prescribed medications for prophylaxis of non-immune travellers are doxycycline, atovaquone-proguanil, and mefloquine [4]. The tetracycline class of antibiotics, including doxycycline, have broad spectrum antimicrobial activity, including against protozoan parasites [5], through the inhibition of protein translation due to binding of the prokaryotic-like 70S ribosome inside the essential apicoplast organelle. This mechanism is thought to contribute to the delayed death effect in blood stages seen with this drug class. The use of tetracyclines to treat patients with uncomplicated *P. falciparum* and *P. vivax* malaria infections dates to the 1950s [6–8], while doxycycline has been used for prophylaxis since 1985 [9] and was formally approved by the US Food and Drug Administration (FDA) for prophylaxis in 1994 [10]. Available treatments include quinine derivatives (chloroquine and mefloquine), 8-aminoquinoline compounds (primaquine and tafenoquine), antifolates (proguanil), and artemisinin compounds. Artemisinin-based combination therapy (ACT) has revolutionized malaria treatment as it is highly effective against both *P. falciparum* and *P. vivax.*

Increasing rates of drug resistance and significant side effects/contraindications of existing antimalarial regimens demonstrate the need for new agents. For example, 8-aminoquinolines have limited use due to the severe haemolytic anaemia observed in people with glucose-6-phosphate dehydrogenase (G6PD) deficiency, mandating genetic testing [11]. Treatment with doxycycline can lead to clinical and prophylactic failure due to inadequate dosing and poor compliance due to side effects [12, 13]. Increased use of artemisinin has dramatically reduced the global disease burden of malaria; however, artemisinin resistance, characterized by slow parasite clearance due to mutations in the Kelch protein, was initially detected in Cambodia and has since spread across Southeast Asia [14], with some resistant cases noted in Africa [15]. Two drug combinations of artemisinin and its derivates with other antimalarial drugs are now recommended by the WHO in Southeast Asia due to resistance to multiple treatment regimens [11]. By 2021, Kelch mutations and increased parasite clearance time were identified in Uganda and Rwanda, indicating independent emergence of resistance and local spread of artemisinin resistant isolates [16, 17]. There is a growing emergence of *Plasmodium* that are resistant to other standard-of-care (SOC) drugs as well, including doxycycline, atovaquone-proguanil, and mefloquine. For these reasons, the need to develop safe and affordable drugs for treatment and prevention of malaria is still present.

An important strategy to fill these unmet needs is to identify and repurpose US FDA-approved therapeutics that demonstrate antimalarial efficacy in order to streamline development [18]. For example, in a recent phase 2 trial, the US FDA-approved chemotherapeutic imatinib was added to the SOC for *P. falciparum* malaria in adult male patients only (due to unknown effects on pregnancy). This trial showed promising results: no increase in number or severity of adverse events, significantly accelerated decline in parasite density and pyrexia, and no delayed parasite clearance [19]. Antibiotics, including macrolides, quinolones, lincosamides, and tetracyclines are of particular interest as alternative therapies because they exert a different mode of action than current non-antibiotic antimalarials and can be used to treat concomitant bacterial infections [20].

Omadacycline is a first-in-class aminomethylcycline antibiotic of the tetracycline class, approved in the US for the treatment of adults with community-acquired bacterial pneumonia and acute bacterial skin and skin structure infections with both oral (PO) and intravenous (IV) formulations [21, 22]. Omadacycline is designed to overcome the most clinically relevant bacterial tetracycline class resistance mechanisms including efflux pumps and ribosomal protection proteins [22]. For example, omadacycline maintains activity against pathogens expressing the ribosomal protection protein Tet(M), which contributes to doxycycline resistance [23]. In this study, the activity and efficacy of omadacycline, alongside standard comparators, was evaluated in in vitro and in vivo models of *Plasmodium* spp. infection.

## Methods

### Ethical approval for animal use

The animal protocols for this study were approved by the Walter Reed Army Institute of Research Institutional Animal Care and Use Committee in accordance with national and Department of Defense guidelines. Research was conducted in an AAALAC accredited facility in compliance with the Animal Welfare Act and other federal statutes and regulations relating to animals and experiments involving animals and adheres to principles stated in the Guide for the Care and Use of Laboratory Animals.

### Animals

Female albino C57BL/6 mice weighing 18–22 g purchased from Charles River Laboratories (Wilmington, MA) were used to conduct in vivo efficacy studies. The mice were left to acclimatize for 7 days prior to the beginning of research studies. All mice were assigned a study number with an individual ear tag. Cards attached to each mouse cage were also used to identify the study groups. The mice were acclimatized for 7 days. Mice were housed in a designated room with food and water supplied ad libitum and a 12:12 h light: dark cycle. Throughout the study, animals that met the criteria for euthanasia, such as a drop in body temperature of ≥ 3 °C compared to the baseline, weight loss ≥ 15%, etc., were considered to have parasite recrudescence and were euthanized to minimize unnecessary pain or distress.

### Parasites

Four well-characterized laboratory standard clones (D6, C235, W2, and C2B) of *P. falciparum* with differing susceptibility patterns to known antimalarials were evaluated for susceptibility to doxycycline and omadacycline. D6 is a clone of Sierra Leone I/CDC [24, 25]. D6 is considered a sensitive clone, with susceptibility to chloroquine (CQ), quinine, pyrimethamine (PYR), and atovaquone (ATOV) [26]. D6 also has natural reduced susceptibility to mefloquine (MFQ) [27]. Tm91-C235 (C235) is a RII mefloquine treatment failure isolate from a study conducted at the Bangkok Hospital for Tropical Diseases [28]. C235 is sensitive to ATOV and is resistant to CQ, MFQ, quinine, and dihydrofolate reductase (DHFR) and dihydropteroate synthase (DHPS) inhibitors such as PYR and sulfadoxine, respectively. W2, a multidrug resistant isolate, is a clone of Indochina III/CDC [24, 25, 28]. This clone is resistant to CQ, PYR, quinine, and sulfadoxine, and is sensitive to MFQ and ATOV. Tm90-C2B (C2B) was isolated from a patient in Thailand after an R1-type recrudescence during a Phase 2 atovaquone study [29]. C2B is resistant to CQ, MFQ, quinine, PYR and ATOV.

*Plasmodium cynomolgi bastianellii* (B strain) sporozoites derived from *Anopheles dirus* mosquitoes were used for the in vitro liver stage assessment and were supplied by the Armed Forces Research Institute of Medical Science (AFRIMS). Luciferase and GFP-expressing *P. berghei* ANKA parasite strain (MRA-868) was obtained from Malaria Research and Reference Reagent Resource Center [30] for use in the in vivo murine *P. berghei* infection model (BEI Resources).

### Drugs and drug preparation

For in vivo efficacy studies, drugs tested were obtained from the following vendors: Omadacycline, 100 mg/vial for injection (Paratek Pharmaceuticals, Inc., King of Prussia, PA); Tafenoquine and 4-Methyl primaquine (WR238605-21 and WR181023-7, respectively; WRAIR, Experimental Therapeutics Branch Repository, Specs US Compound Management Facility, Cumberland, MD); Doxycycline hyclate catalog #D989 (Sigma Aldrich, St. Louis, MO). Drugs were formulated as follows: omadacycline in 0.9% sodium chloride (Baxter Healthcare Corporation, Deerfield, IL); Tafenoquine in 2% Tween-80 (Sigma Aldrich, lot #MKBL8793V), 98% cell culture grade water (Corning Inc, Corning, NY, lot #13121022); 4-Methyl-Primaquine and doxycycline in cell culture grade water (Corning, lot #13121022). Vehicle control (VC) consisted of 0.9% sodium chloride (Baxter Healthcare Corporation, Deerfield, IL).

For in vitro assays, drugs tested were obtained from the following vendors: Omadacycline (catalog # HY-14865 MedChemExpress, Monmouth Junction, NJ and Paratek Pharmaceuticals, Inc., King of Prussia, PA for the *P. falciparum* and *P. cynomolgi* assays, respectively); Tafenoquine and doxycycline (WR238605-21 and WR100553-30, respectively; WRAIR, Experimental Therapeutics Branch Repository, Specs US Compound Management Facility, Cumberland, MD); Chloroquine catalog (#C6628-25G, Sigma Aldrich, St. Louis, MO). All compounds were first dissolved in 100% dimethylsulfoxide (DMSO) for a stock concentration of 20 mM.

### In vivo murine *P. berghei* infection and liver and blood stage efficacy

The efficacy of omadacycline and comparators doxycycline, tafenoquine, primaquine, and vehicle control was evaluated as prophylaxis against malaria using the In vivo Imaging System (IVIS) [31]. Mice were infected intravenously with 10^4^ luciferase-expressing *P. berghei* sporozoites (ANKA strain) on Day 0 via tail vein injection [31]. All test compounds were administered once per day on Day −1, Day 0, and Day 1.

Omadacycline and its vehicle control were administered intraperitoneally (IP) at 5, 10, 20 or 30 mg/kg since omadacycline lacks oral bioavailability in rodents [32], while doxycycline, tafenoquine, and primaquine were administered via oral gavage (120, 10, and 5 mg/kg, respectively). Five mice were included per treatment group, except for tafenoquine, which had only four mice due to an unexpected animal death attributed to a gavage error.

Causal prophylaxis was determined by assessing the percent reduction of bioluminescence signal emitted in the liver area at 48 h post-infection, while the early blood stage was assessed at 72 h by examining signal emitted from the whole animal. Infection reduction was assessed by comparing the bioluminescence signal emitted by the treated groups with the signal emitted by the vehicle control treated group at that time point. Later blood stage parasitaemia was determined via flow cytometry starting on day 6 post-infection and continued for up to 30 days. In this model, an animal is considered cured if liver stage bioluminescence signal is below the IVIS limit of detection (LOD) at 48 h post infection and blood stage parasitaemia is below the flow cytometer LOD at 30 days post infection.

### In vivo bioluminescence imaging of mice infected with luciferase—expressing *P. berghei* parasites

Luciferin (D-Luciferin potassium salt, Xenogen, CA and Gold Biotechnology, St. Louis, MO), the luciferase substrate, was inoculated IP at a concentration of 200 mg/kg into female albino C57BL/6 mice using a 27½ gauge needle, 10 min before bioluminescence analysis. Animals were anaesthetized in a 2.5% isoflurane atmosphere (MWI Veterinary Supply, Harrisburg, PA) for 5 min and maintained in the imaging chamber for analysis. Emitted photons were collected by auto acquisition with a charge coupled device (CCD) camera (Perkin Elmer Spectrum IVIS) using the medium resolution (medium binning) mode. Analysis was performed after defining a region of interest that delimited the surface of the affected area. Whole-body total photon emission was quantified with Living Image software (Xenogen Corporation, Alameda, CA), and results were expressed in numbers of photons/second. Causal prophylaxis was determined by assessing the percent reduction of bioluminescence signal emitted in the liver area at 48 h post-infection, while the early blood stage was assessed at 72 h by examining signal emitted from the whole animal. Infection reduction was assessed by comparing the bioluminescence signal emitted by the treated groups with the signal emitted by the vehicle control treated group at that time point.

### Parasitaemia measurements

Parasitaemia was measured via flow cytometry as previously described [33, 34]. In brief, 3 µL of blood was obtained via the mouse tail vein and transferred into 0.3 mL of 1% heparinized isotonic PBS buffer. One mL of 0.04% of glutaraldehyde was added to fix each sample, and samples were incubated for 60 min at 4 °C followed by centrifugation at 450 g, 5 min at room temperature (RT). The supernatant was removed by aspiration, and the cells were resuspended in 0.5 mL PBS buffer supplemented with 0.25% (v/v) Triton X-100 (Sigma-Aldrich St Louis, MO), incubated for 10 min at RT, and centrifuged again at 450 g, 5 min at RT. After centrifugation, the permeabilized cells were suspended in 0.5 mL of RNase (Sigma-Aldrich St Louis, MO) at 1 mg/mL concentration and incubated for at least 2 h at 37 °C to ensure complete digestion of reticulocyte RNA. 20ul of YOYO-1 dye (Invitrogen Corp. Carlsbad, CA) at a concentration of 2500 ng/mL was added to the 0.5 mL sample volume to create a final dye concentration of 100 ng/mL of YOYO-1. Parasitaemia was quantified using an FC500 MPL flow cytometer (Beckman Coulter, Fullerton, CA). The green photomultiplier tube and filter setting was used for these studies (520–555 nm filter settings). Infected erythrocytes, uninfected erythrocytes, and leukocytes were gated on logarithmic forward/side dot plots. The detection limit for a negative flow cytometry result was defined as the failure to observe a parasite after examination of 1000 red blood cells (i.e., 0.1% parasitaemia, which is the lowest value indicated by the flow cytometry instrument).

### *Plasmodium falciparum* asexual blood stage assay

*Plasmodium falciparum* laboratory clones (D6, C235, W2, and C2B) were kept continuously in long-term cultures as described [26]. In vitro asexual blood stage antiplasmodial activity was determined by the Malaria SYBR Green I-based Fluorescence (MSF) assay described previously [26] with minor modifications for use of 384-well microtiter plates. Briefly, 20 mM stock compounds were diluted in basic culture medium [(BCM); RPMI 1640 containing 2000 µg/mL D-(+)-Glucose, 5950 µg/mL 4-(2-hydroxyethyl)−1-piperazineethanesulfonic acid (HEPES)] to a 200 µM intermediary starting concentration for subsequent serial dilutions. Pre-filled 384-well microtiter plates containing 4.25 µL volume per well were prepared containing duplicate 12-point, twofold serial dilutions of each compound. Final concentration range tested for the compounds was 20–0.01 µM. *P. falciparum* parasites in ring or early-trophozoite stages were diluted in BCM to a starting parasitaemia of 0.15% and 2% haematocrit in 38 µL volume per well was added to the pre-filled drug assay plates using the Tecan Freedom EVO liquid handling system (Tecan USA, Inc.). This resulted in a final DMSO concentration of 0.02% or less. Plates were incubated at 37 °C in a humidified atmosphere of 5% CO_2_, 5% O_2_, and 90% N_2_ for 96 h. The assay was extended to 96 h in order to cycle through two lifecycles of the parasites due to the delayed death typically seen with antibiotics. A volume of 38 µL of lysis buffer, consisting of 20 mM Tris–HCl, 5 mM ethylenediaminetetraacetic acid (EDTA), 1.6% Triton-X, 0.016% saponin, and SYBR Green I dye at 20X concentration was added to the assay plates for a final SYBR Green concentration of 10X. Plates were incubated in the dark for 24 h before assessing relative fluorescence units (RFU) using the Tecan Infinite 200 Pro plate reader (Tecan USA, Inc.).

### *Plasmodium cynomolgi* liver stage assay

The activity of doxycycline and omadacycline was evaluated in the *P. cynomolgi* liver stage (*Pc*LS) assay using primary simian hepatocytes infected with *P. cynomolgi* sporozoites [35–38]. Cryopreserved primary cynomolgus monkey hepatocytes and hepatocyte culture medium (HCM) (InVitroGro CP medium) were obtained from BioIVT, Inc. (Baltimore, MD, USA) and thawed following manufacturer recommendations. 24–48 h after plating hepatocytes, freshly dissected sporozoites were introduced and allowed to infect overnight. A single plate of infected hepatocytes was treated with compound for 3 consecutive days in triplicate 12-point threefold serial dilutions of each compound suspended in 100% DMSO. Compounds were transferred to infected hepatocytes using a pin tool (V&P Scientific, LLC), for a final concentration range of 20–0.0001 µM and 0.1% DMSO. Following drug administration, remaining parasites were allowed to develop until 8 days post-infection (dpi) when cells were fixed, labeled via Indirect Fluorescent Antibody (IFA) assay, and imaged on the Operetta CLS Imaging System (Perkin Elmer, MA, USA) as previously described [38–40]. Parasite quantities and hepatocyte counts were computer tabulated by previously established object detection methods [40] using Harmony 4.9 software (Perkin Elmer, MA, USA). Size and morphological features were used to classify detected schizont and hypnozoite forms as previously described [40]. Raw counts were normalized to objects classified in stained uninfected controls.

## Data analysis

For in vivo imaging studies, GraphPad Prism 10.1.2 was used to perform analysis of variance (ANOVA) with multicomparison test (Tukey/Duncan test) to assess the difference between bioluminescence signal emitted from the liver and whole-body area. Infection reduction was assessed by comparing the bioluminescence signal emitted by the treated groups with the signal emitted by the vehicle control treated group at that time point. The value emitted by clear skin (1.59E + 05 photons/sec) was used for animals in which no bioluminescence signal was detected in the body area. Kaplan–Meier survival curves were constructed by using GraphPad Prism 10.1.2 and analysed via a Log-rank (Mantel-Cox) test (p < 0.5) in SigmaPlot (V.X), followed by individual pair-wise comparisons between subject groups using a Bonferroni-corrected threshold for multiple comparisons.

For both the MSF and *Pc*LS assays, the percentage of inhibition (PI) were calculated in Python using Eq. 1, using RFU measurements or stain control normalized parasite quantities (hypnozoite and schizont counts), respectively. Drug induced hepatocyte death (toxicity) was also calculated with Eq. 1, using hepatocyte nuclei counts as previously described [40]. Changes in the number of hepatic nuclei with respect to drug concentration were taken as a measure of drug cytotoxicity (cell death or inhibition of proliferation). For curve fitting, negative inhibition (counts or RFU greater than that of average negative control) values were clipped to 0. Each plate included negative (DMSO) and assay specific positive controls (MSF: chloroquine; *Pc*LS: tafenoquine). The 50% inhibitory concentration (IC_50_) values were determined using a 12-point, threefold dilution format and a Python-adapted grid algorithm for fitting PI vs concentration to a four-parameter logistic function [41–45].

1 $$\varvec{PI}\, = \,~\mathbf{100}\,*\,\left( {\mathbf{1}\, - \,\varvec{\left( {\frac{{xy}}{{Mean_{{neg}} }}} \right)}} \right)$$

## Results

### In vivo efficacy of omadacycline as causal prophylaxis against *P. berghei*

**Fig. 1:**
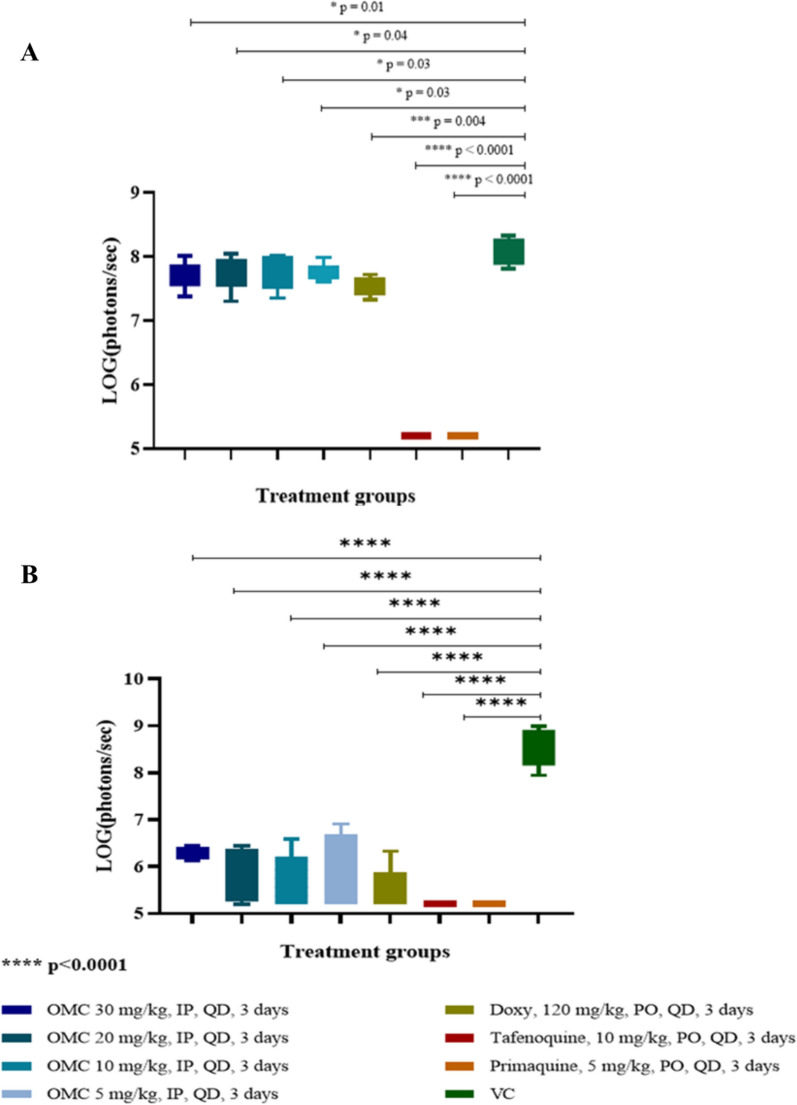
Suppression of *P. berghei* parasite luminescence signal by omadacycline in **A** liver (48 h) and **B** early blood stage (72 h) in a murine causal prophylaxis assay. Each timepoint represents the mean ± SEM bioluminescence signal emitted by **A** liver and **B** whole body area for n = 5 mice (all treatment groups, except for the Tafenoquine treated group (n = 4)). OMC = omadacycline; Doxy = doxycycline; VC = vehicle control; IP = intraperitoneal; PO = oral; QD = once daily. Note: The value emitted by clear skin [1.59E+05 photons/sec (LOG = 5.2)] was used for animals in which no bioluminescence signal was detected in the body area

**Fig. 2:**
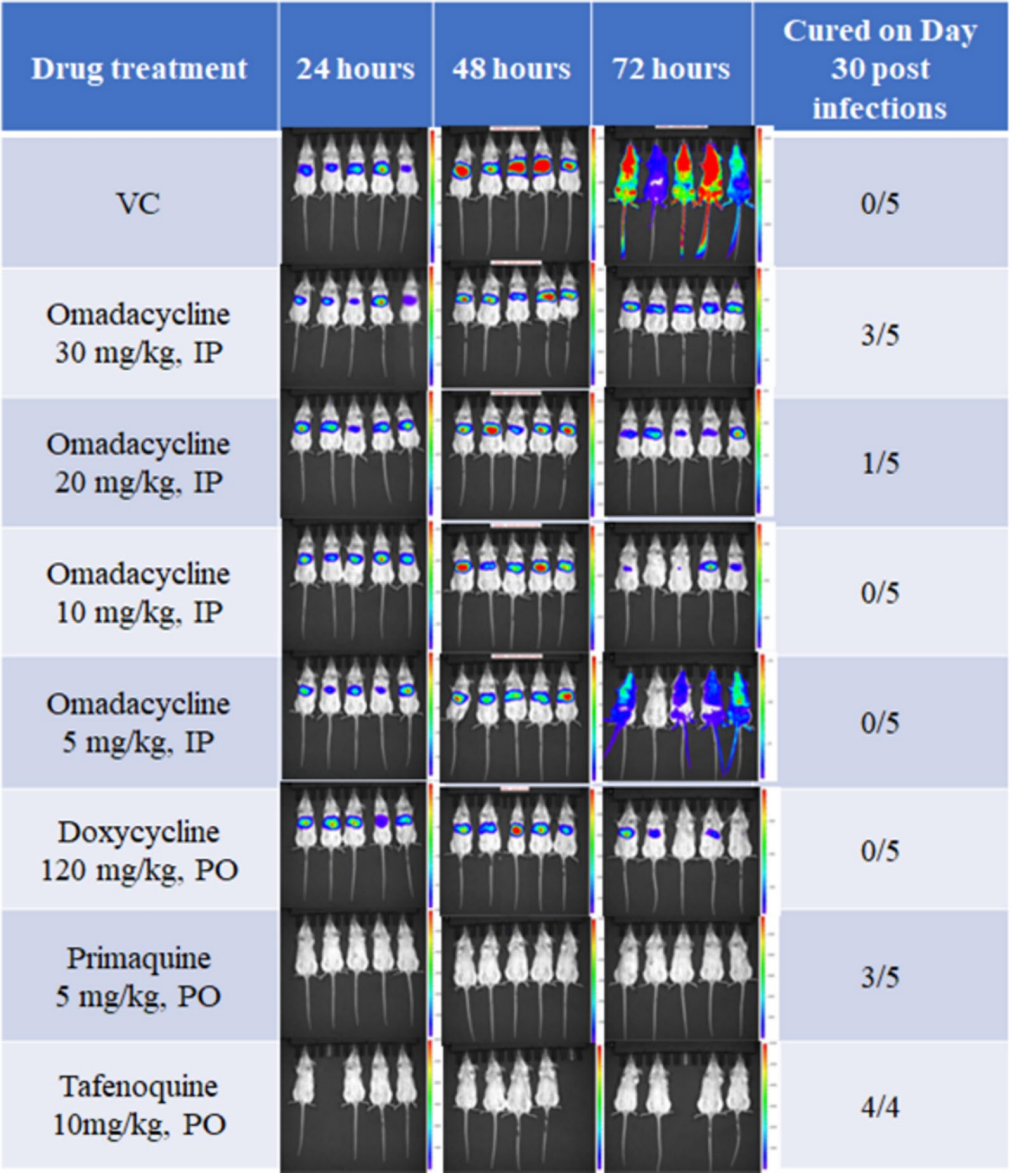
Visualization of *P. berghei* parasite luminescence signal in the causal prophylaxis assay. The photon intensity numbers on the body surface represent malaria parasites in liver and blood. The brighter areas of the image (red or bright yellow) show a higher parasite load compared to the areas with a dimmer green or blue color, where fewer photons were detected. Lack of bioluminescence light on the body surface represents total lack of parasite load (malaria free) or a parasite load below the limit of detection. VC = vehicle control; IP = intraperitoneal; PO = oral

All omadacycline treated groups suppressed luminescent signal (which represents the parasite load) in the liver of mice infected with luciferase-expressing *P. berghei* sporozoites, when compared to the vehicle control (Figs. 1A and 2) at 24 and 42 h post infection. This suppression was similar to doxycycline and consistent with the in vitro schizonticidal activity observed. The positive controls tafenoquine and primaquine, 8-aminoquinolines with known efficacy against liver-stage malaria, reduced the bioluminescence signal below the LOD (Figs. 1A, B, and 2).

Control mice displayed bioluminescent signal visible over their entire body area at 72 h post infection, consistent with parasite migration from the liver to blood-stage infection (Figs. 1A and 2). All doses of omadacycline tested significantly decreased bioluminescent signal compared to control mice (Figs. 1B and 2), indicating omadacycline was acting in a suppressive prophylactic manner. A similar result was seen with doxycycline and this data is in line with the observed in vitro activity. No significant signal above background was detected in tafenoquine or primaquine treated mice.

**Fig. 3:**
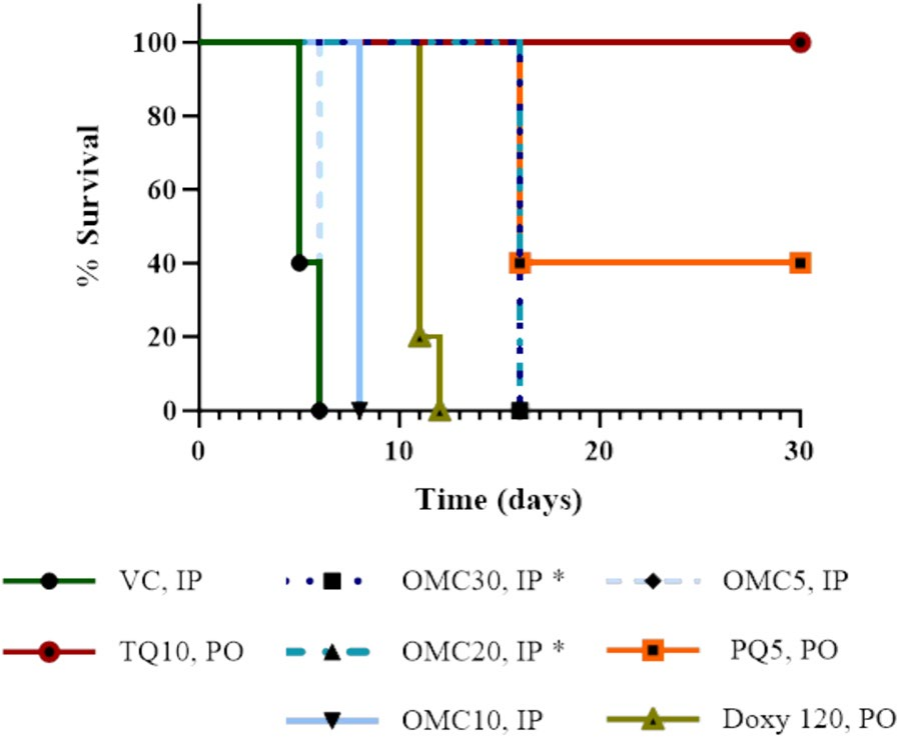
Omadacycline increased animal survival during blood stage *P. berghei* malaria infection in a dose-dependent manner. The effects of omadacycline on mouse survival after *P. berghei* infection were determined via the Kaplan–Meier method. All treatment groups were significantly more effective than VC (p < 0.05). *OMC30 and OMC20 mg/kg (but not OMC10 and OMC5) significantly extended animal survival time compared to Doxycycline 120 mg/kg (p < 0.05). TQ was significantly more effective than all other treatment groups (p < 0.05). OMC = omadacycline (OMC30 mg/kg = dark blue squares and dots, OMC20 mg/kg = turquoise lines and black triangles; OMC10 mg/kg = light blue line and black triangles, OMC5 mg/kg = light blue lines and black diamonds); Doxy = doxycycline (dark olive triangles); tafenoquine (dark red circle); primaquine (bright orange squares); VC = vehicle control (dark green circles); IP = intraperitoneal; PO = oral

All control animals succumbed to infection on day 6, while the omadacycline treated mice showed a dose dependent increase in survival (Fig. 3). In fact, 1/5 (20%) and 3/5 (60%) mice treated with omadacycline at doses of 20 and 30 mg/kg/day, respectively, survived to day 30 with no detectable parasitaemia. In contrast, parasitaemia was detectable in some 20% (1/5) doxycycline treated mice at day 8 post-infection (data not shown) and all mice in that group were euthanized by day 15 (Fig. 3). Only tafenoquine met our criteria for animal cure at the end of the 31-day period. All treatment groups were significantly more effective than the vehicle control, while the 30 and 20 mg/kg omadacycline (but not 5 and 10 mg/kg) significantly extended animal survival time compared to doxycycline. Historically, a dose of 5 mg/kg primaquine resulted in total cure, but in this study, only 3/5 (60%) of mice treated with this dose were parasitaemia free at 30 days. Although primaquine was able to suppress luminescent signal below the LOD of the assay, it was not able to completely eradicate the infection, leading to the delayed development of active blood stage infection.

### In vitro activity of omadacycline against blood stage *P. falciparum* and liver stage *P. cynomolgi* schizonts

**Fig. 4:**
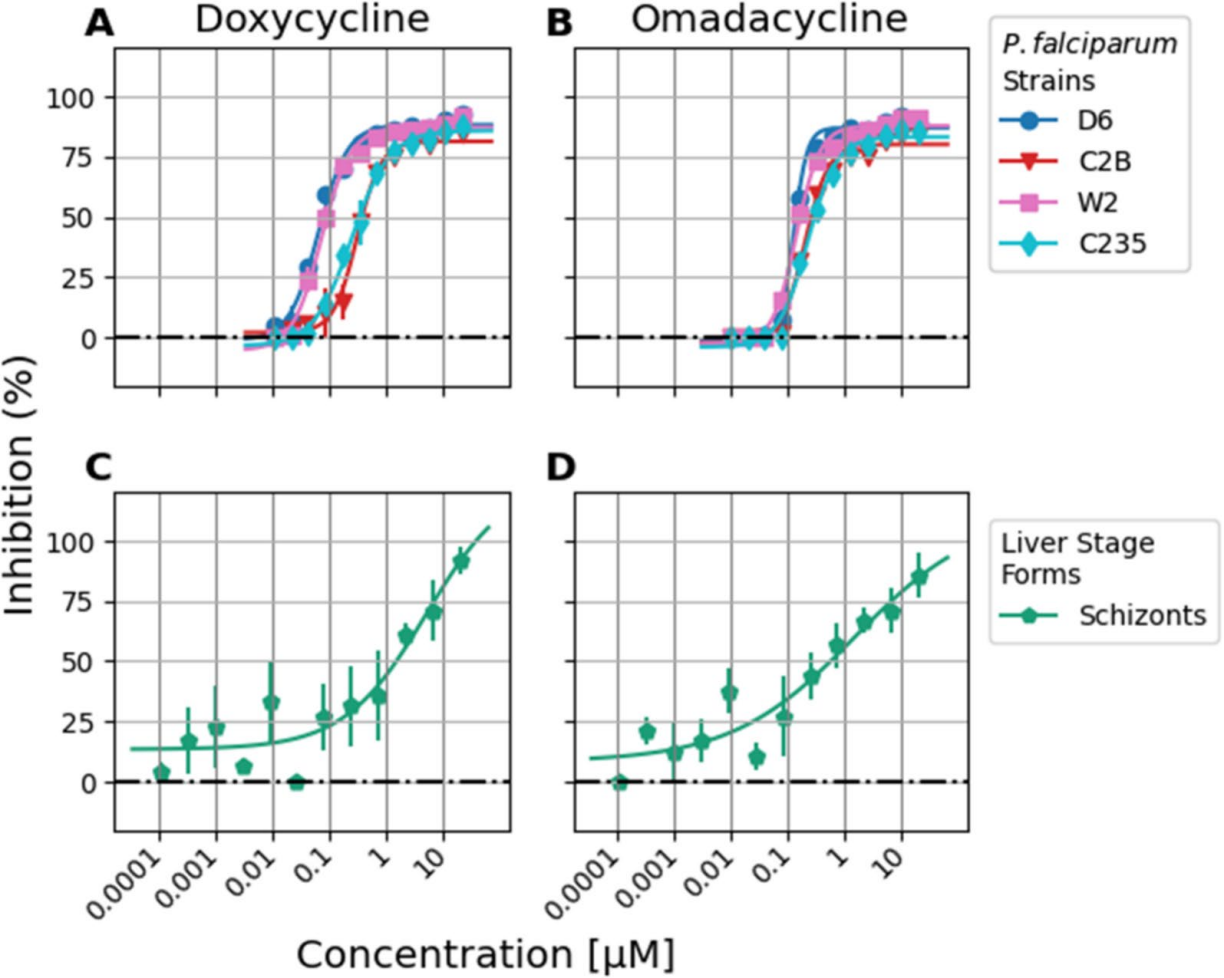
IC_50_ curves for doxycycline and omadacycline in vitro anti-*Plasmodium* activity. Plots display log micromolar concentration vs percent inhibition, with black dashed dotted line as 0%, and error bars representing standard error mean of single assay replicates. **A**, **B** *P. falciparum* blood stage SYBR Green assay. **C**, **D** Liver stage *P. cynomolgi* assay

Doxycycline distribution of IC_50_ values were 0.08 and 0.09 µM against D6 and W2 strains, respectively, and had higher IC_50_ values of 0.41 and 0.34 µM against the resistant isolates C2B and C235, respectively. (Fig. 4A, Table 1). Omadacycline IC_50_ values were 0.14 and 0.17 µM against D6 and W2, respectively, and were slightly higher against the multi-drug resistant strains with IC_50_ values of 0.24 and 0.30 µM against C2B and C235, respectively (Fig. 4B, Table 1).

**Table 1:** IC_50_ values and maximum inhibition for *Plasmodium falciparum* in SYBR Green blood stage assay and *Plasmodium cynomolgi* in liver stage prophylactic assay

*P. falciparum* (Asexual Blood Stage)^a^						
Clone	Doxycycline		Omadacycline		Chloroquine	
	IC_50_ (µM)	Max inhibition (%)	IC_50_ (µM)	Max inhibition (%)	IC_50_ (µM)	Max Inhibition (%)
D6	0.08	92	0.14	89	0.01	93
W2	0.09	92	0.17	91	0.31	91
C2B	0.39	85	0.26	85	0.21	86
C235	0.35	87	0.30	85	0.10	90

*P. cynomolgi* (Liver Stage)^b^						
Forms	Doxycycline		Omadacycline		Tafenoquine	
	IC_50_ (µM)	Max Inhibition (%)	IC_50_ (µM)	Max Inhibition (%)	IC_50_ (µM)	Max Inhibition (%)
Hypnozoite	> 20	3.4	> 20	0.0	1.6	100
Schizont	1.5	92	0.55	85	0.23	100
Toxicity	> 20	0.55	> 20	2.6	13	77

Maximum inhibition represents the mean percent inhibition value at the highest concentration tested (20 µM). Toxicity was measured as changes in hepatic nuclei counts

^a^IC_50_ values are the average of a single biological replicate for each clone with in-plate duplicates of compound titrations

^b^IC_50_ values are the average of a single biological replicate with in-plate triplicates of compound titrations

As expected for tetracycline class drugs, neither doxycycline nor omadacycline had an appreciable effect on dormant hypnozoite counts nor on the host hepatocyte toxicity as measured by Hoechst stain. The IC_50_ for anti-schizont activity was 1.5 µM for doxycycline and 0.55 µM for omadacycline (Fig. 4C and D, Table 1). Tafenoquine was used as the assay positive control which showed IC_50_ values of 1.64 µM against hypnozoites and 0.23 µM against schizonts.

## Discussion

Tetracyclines have proven to be valuable drugs for antimalarial therapy and their continued use underscores the utility of screening tetracyclines for improved activity against malaria. In these studies, we examined the activity and efficacy of a member of the tetracycline class, omadacycline, in well characterized in vitro and in vivo models, respectively, of *Plasmodium* infection. After a 96-h incubation, omadacycline demonstrated similar in vitro activity against both drug sensitive and resistant isolates of *P. falciparum* (IC_50_ range of 0.14–0.30 µM), while doxycycline was more active against the drug sensitive isolates compared to the multi-drug resistant isolates (IC_50_ range of 0.08–0.41 µM)*.* The in vitro concentrations of doxycycline and omadacycline tested here are within the range of concentrations observed in human serum with US FDA-approved oral dosing regimens: doxycycline 100 mg oral dose, maximum serum concentration (C_max_) = 2.8 µg/mL (5.5 µM), 200 mg oral dose, C_max_ = 4.3–9.3 µg/mL (8.4–18.1 µM) [10] and omadacycline 300 mg oral dose, C_max_ at steady state = 9.5 µg/mL (13 µM) steady state [21]). The drug concentrations reached in serum after oral dosing for omadacycline and doxycycline reached an IC_50_ of ≥ 75% for the parasite strains tested here but the clinical relevance of this is not yet known. Neither drug was effective against liver stage *P. cynomolgi* hypnozoites, while both showed partial activity against schizonts.

Preliminary studies have reported antimalarial activity for two other newer tetracycline class members, tigecycline and eravacycline. Tigecycline displayed in vitro antiplasmodial activity against *P. falciparum* with IC_50_s ranging from 0.20 to 0.70 µM [46–49], depending on the assay and isolate used. Eravacycline also demonstrated in vitro activity against *P. falciparum* (IC_50_s ranged from 0.014–2.0 µM, depending on the isolate and assay length) [49]. Although tigecycline and eravacycline show activity in in vitro studies, a major drawback is that they are only approved for parenteral administration due to poor oral bioavailability and thus would not be an option for prophylaxis or during travel to remote areas.

Of significance, the in vitro activity of omadacycline was confirmed in vivo against *P. berghei* in a murine causal prophylaxis model. IP administration of omadacycline resulted in a slight suppression of luminescence at 48 h post infection during liver stage parasite growth, consistent with the activity observed on *P. cynomolgi* schizont development. However, all doses of omadacycline tested demonstrated significant suppression of whole-body luminescence (blood stage) at 72 h post-infection. At this time point, parasites have started exiting the liver and have begun their erythrocytic replication cycle. In addition, omadacycline treatment resulted in a dose-dependent increase in parasitaemia-free survival to the end of study, with survival of 20% (1/5) of mice observed in the 20 mg/kg treatment group and 60% (3/5) of mice observed in the 30 mg/kg treatment group. In contrast, 0% (0/5) survival was observed for mice in the 120 mg/kg doxycycline treatment group. The 20 mg/kg dose of omadacycline administered to mice in this study most closely approximates the human exposure based on the current US FDA approved dosing regimens [32]. Published PK data suggests that the doxycycline dose evaluated in mice in this study would result in exposures that are approximately twofold higher than the human equivalent dose [50]. While further investigation is needed to define the exposures in mice and the clinical relevance when extrapolated to humans, these results are consistent with omadacycline acting as a suppressive prophylactic against *Plasmodium* in mice, similar to doxycycline [5, 8].

Tigecycline reportedly showed limited in vivo blood stage efficacy in *P. berghei*-infected mice [51], with only the highest dose (100 mg/kg/day IP for four days) resulting in cure on day 28. Although there are differences in the dosing and *P. berghei* strain used between these two studies, we speculate that a modestly higher dose of omadacycline would likely result in a 100% cure rate in our murine prophylactic model and warrants further investigation, especially in comparison to tigecycline in the in vivo blood stage assay [51].

There are several limitations of this work, including the small number of isolates evaluated in vitro and a lack of data surrounding other species of *Plasmodium*, including ACT resistant isolates. In addition, omadacycline was evaluated alone, and therefore future studies may be warranted to investigate the activity of omadacycline in combination with other antimalarials since two- and three-drug treatment combinations are becoming more common. The current causal prophylactic study was not designed to assess the in vivo blood schizonticidal activity of omadacycline. Additional testing after injecting blood-stage, rather than sporozoites, *P. berghei* to mice (i.e., Thompson test) is needed. Another limit of this study includes the limited dosing period in the mouse; extension of the dosing period to mimic human dosing regimens would provide a greater understanding of how this data relates to efficacy in humans. A pre-exposure prophylaxis study would also be of interest as doxycycline is mainly prescribed for prophylaxis. In addition, PK analysis of omadacycline and the comparator drugs was not performed in this work and extrapolations must be made to hypothesize the expected exposure based on available historical data.

Doxycycline remains highly effective for *P. falciparum* prophylaxis although tetracycline-resistant mutants can be isolated after repeated exposure in mice, and several genetic markers of reduced susceptibility to doxycycline have been identified in clinical isolates (e.g., *pftetQ* and *pfmdt, Plasmodium* analogues of bacterial transporters and ribosomal genes involved in tetracycline resistance) [52–55]. Additionally, there is at least one documented case of doxycycline prophylaxis failure in humans [55]. Based on the mechanism of action of omadacycline and its ability to overcome common tetracycline resistance mechanisms in bacteria, omadacycline may retain activity against tetracycline-resistant *Plasmodium*. The antiplasmodial activity of tetracyclines has been shown to be effective during the blood stages of malaria infection (schizonticidal agent), with a slow-acting mechanism of action [20]. Prophylactic use of doxycycline requires daily oral administration while one is present in endemic areas and requires continued daily dosing after returning to non-endemic areas for an additional 28 days to allow time for parasites to emerge from the liver. However, varying side effects of the doxycycline prophylactic regimen have been reported, including gastrointestinal effects, mouth ulcers, and phototoxicity, which can lead to poor compliance and a loss of efficacy [10]. Military deployment or civilian exposure to areas endemic with *P. vivax* would also include treatment with an antimalarial effective against hypnozoites, such as primaquine or tafenoquine [11, 56] to prevent relapsing disease upon return to non-endemic areas. Omadacycline is formulated and approved for oral administration [21] and is well tolerated, with gastrointestinal effects being the most commonly reported side effect. In addition, although phototoxicity is a safety concern for all tetracyclines, omadacycline did not meet criteria for a phototoxic response in mammalian cell assays in vitro [57]. Omadacycline is not indicated for use in pregnant women and children under 8 (similar to doxycycline), or adolescents under 18 [10, 21]. However, at the time of this writing, a Phase 1 clinical study to evaluate the PK of IV and PO omadacycline in children and adolescents with suspected or confirmed bacterial infections is ongoing (NTC05217537).

## Conclusions

These studies demonstrate the efficacy of omadacycline in a murine prophylaxis model of *P. berghei* infection as well as in vitro activity against *Plasmodium* isolates, regardless of drug resistance. Unlike other newer tetracycline class drugs, omadacycline is approved for oral administration and is without the photosensitivity liability of doxycycline. These results warrant additional research to further understand the potential utility of omadacycline as prophylaxis for malaria.

## Data Availability

The datasets used and/or analyzed during the current study are available from the corresponding author on reasonable request.

## Abbreviations

AAALAC: Association for the Assessment and Accreditation of Laboratory Animal Care
ACT: Artemisinin combination therapies
ATOV: Atovaquone
AUC: Area under the curve
CCD: Charge coupled device
CDC: Centers for Disease Control
C_max_: Maximum serum concentration
CQ: Chloroquine
DHFR: Dihydrofolate reductase
DHPS: Dihydropteroate synthase
DMSO: Dimethylsulfoxide
DOXY: Doxycycline
EDTA: Ethylenediaminetetraacetic acid
FDA: Food and Drug Administration
GFP: Green fluorescent protein
G6PD: Glucose-6-phosphate dehydrogenase
HCM: Hepatocyte culture medium
IACUC: Institutional Animal Care and Use Committee
IC_50_: 50% Inhibitory concentration
IFA: Indirect fluorescent antibody
IP: Intraperitoneal
IV: Intravenous
IVIS: In vivo imaging system
LOD: Limit of detection
MFQ: Mefloquine
MSF: Malaria SYBR Green I-Based Fluorescence
MSMR: Medical Surveillance Monthly Report
OMC: Omadacycline
PK: Pharmacokinetic
PO: Oral
PYR: Pyrimethamine
RFU: Relative fluorescence units
RT: Room temperature
SOC: Standard of care
US: United States
VC: Vehicle control
WHO: World Health Organization

## References

[1] WHO. World Malaria Report. addressing inequity in the global malaria response. Geneva: World Health Organization; 2024. p. 2024.

[2] Update: Malaria, U.S. Armed Forces, 2018. MSMR. 26:2–7 30807196

[3] Bagcchi S. Locally acquired malaria cases in the USA. Lancet Infect Dis. 2023;23: e401. 37776883 10.1016/S1473-3099(23)00581-9

[4] DeVos E, Dunn N. Malaria prophylaxis. (Updated 2023 Jul 3). In: StatPearls (Internet). Treasure Island (FL): StatPearls Publishing; 2025 Jan. Available from: https://www.ncbi.nlm.nih.gov/books/NBK551639/31869103

[5] Rusu A, Buta EL. The development of third-generation tetracycline antibiotics and new perspectives. Pharmaceutics. 2021;13:2085. 34959366 10.3390/pharmaceutics13122085 PMC8707899

[6] Imboden CA Jr, Cooper WC, Coatney GR, Jeffery GM, Studies in human malaria. Trials of aureomycin, chloramphenicol, penicillin, and dihydrostreptomycin against the Chesson strain of *Plasmodium vivax*. J Natl Malar Soc. 2019;1950(9):377–80. 14804097

[7] Sanchez FR, Casillas J, Paredes M, Velazquez J, Riebeling QB. Terramycin in malaria therapy. Pan Am Med Womans J. 1952;59:10–5. 14920045

[8] Grande EN, Sanchez AR, Sanchez FR. The treatment of malaria with tetracycline. Antibiotic Med Clin Ther. 1956;3:193196. 13355230

[9] Tan KR, Magill AJ, Parise ME, Arguin PM, Centers for Disease Control and Prevention,. Doxycycline for malaria chemoprophylaxis and treatment: report from the CDC expert meeting on malaria chemoprophylaxis. Am J Trop Med Hyg. 2011;84:517–31. 21460003 10.4269/ajtmh.2011.10-0285 PMC3062442

[10] Apotex Inc. Doxycycline hyclate prescribing information. 2018. https://www.apotex.com/products/us/downloads/pre/doxy_imtb_ins.pdf

[11] Alghamdi JM, Al-Qahtani AA, Alhamlan FS, Al-Qahtani AA. Recent advances in the treatment of malaria. Pharmaceutics. 2024;16:1416. 39598540 10.3390/pharmaceutics16111416 PMC11597227

[12] Pang L, Limsomwong N, Singharaj P. Prophylactic treatment of vivax and falciparum malaria with low-dose doxycycline. J Infect Dis. 1988;158:1124–7. 3053925 10.1093/infdis/158.5.1124

[13] Wallace MR, Sharp TW, Smoak B, Iriye C, Rozmajzl P, Thornton SA, et al. Malaria among United States troops in Somalia. Am J Med. 1996;100:49–55. 8579087 10.1016/s0002-9343(96)90011-x

[14] Dondorp AM, Nosten F, Yi P, Das D, Phyo AP, Tarning J, et al. Artemisinin resistance in *Plasmodium falciparum* malaria. N Engl J Med. 2009;361:455–67. 19641202 10.1056/NEJMoa0808859 PMC3495232

[15] Ashley EA, Dhorda M, Fairhurst RM, Amaratunga C, Lim P, Suon S, et al. Spread of artemisinin resistance in *Plasmodium falciparum* malaria. N Engl J Med. 2014;371:411–23. 25075834 10.1056/NEJMoa1314981 PMC4143591

[16] Balikagala B, Fukuda N, Ikeda M, Katuro OT, Tachibana SI, Yamauchi M, et al. Evidence of artemisinin-resistant malaria in Africa. N Engl J Med. 2021;385:1163–71. 34551228 10.1056/NEJMoa2101746

[17] Umulisa N, Venkatesan M, Svigel SS, Zhou Z, Munyaneza T, Habimana RM, et al. Association of Plasmodium falciparum kelch13 R561H genotypes with delayed parasite clearance in Rwanda: an open-label, single-arm, multicentre, therapeutic efficacy study. Lancet Infect Dis. 2021;21:1120–8. 33864801 10.1016/S1473-3099(21)00142-0 PMC10202849

[18] Pushpakom S, Iorio F, Eyers PA, Escott KJ, Hopper S, Wells A, et al. Drug repurposing: progress, challenges and recommendations. Nat Rev Drug Discov. 2019;18:41–58. 30310233 10.1038/nrd.2018.168

[19] Chien HD, Pantaleo A, Kesely KR, Noomuna P, Putt KS, Tuan TA, et al. Imatinib augments standard malaria combination therapy without added toxicity. J Exp Med. 2021;218:e20210724. 34436509 10.1084/jem.20210724 PMC8404470

[20] Pessanha de Carvalho L, Kreidenweiss A, Held J. Drug repurposing: a review of old and new antibiotics for the treatment of malaria: identifying antibiotics with a fast onset of antiplasmodial action. Molecules. 2021;26:2304. 33921170 10.3390/molecules26082304 PMC8071546

[21] Nuzyra Full Prescribing Information. Paratek Pharmaceuticals, Inc

[22] Tanaka SK, Steenbergen J, Villano S. Discovery, pharmacology, and clinical profile of omadacycline, a novel aminomethylcycline antibiotic. Bioorg Med Chem. 2016;24:6409–19. 27469981 10.1016/j.bmc.2016.07.029

[23] Macone AB, Caruso BK, Leahy RG, Donatelli J, Weir S, Draper MP, et al. In vitro and in vivo antibacterial activities of omadacycline, a novel aminomethylcycline. Antimicrob Agents Chemother. 2014;58:1127–35. 24295985 10.1128/AAC.01242-13 PMC3910882

[24] Teklehaimanot A, Collins WE, Nguyen-Dinh P, Campbell CC, Bhasin VK. Characterization of *Plasmodium falciparum* cloned lines with respect to gametocyte production in vitro, infectivity to Anopheles mosquitoes, and transmission to Aotus monkeys. Trans R Soc Trop Med Hyg. 1987;81:885–7. 3332503 10.1016/0035-9203(87)90336-1

[25] Oduola AM, Weatherly NF, Bowdre JH, Desjardins RE. *Plasmodium falciparum*: cloning by single-erythrocyte micromanipulation and heterogeneity *in vitro*. Exp Parasitol. 1988;66:86–95. 3284758 10.1016/0014-4894(88)90053-7

[26] Johnson JD, Denull RA, Gerena L, Lopez-Sanchez M, Roncal NE, Waters NC. Assessment and continued validation of the malaria SYBR green I-based fluorescence assay for use in malaria drug screening. Antimicrob Agents Chemother. 2007;51:1926–33. 17371812 10.1128/AAC.01607-06 PMC1891422

[27] Rathod PK, McErlean T, Lee PC. Variations in frequencies of drug resistance in *Plasmodium falciparum*. Proc Natl Acad Sci U S A. 1997;94:9389–93. 9256492 10.1073/pnas.94.17.9389 PMC23200

[28] Guan J, Kyle DE, Gerena L, Zhang Q, Milhous WK, Lin AJ. Design, synthesis, and evaluation of new chemosensitizers in multi-drug-resistant *Plasmodium falciparum*. J Med Chem. 2002;45:2741–8. 12061877 10.1021/jm010549o

[29] Canfield CJ, Pudney M, Gutteridge WE. Interactions of atovaquone with other antimalarial drugs against *Plasmodium falciparum* in vitro. Exp Parasitol. 1995;80:373–81. 7729473 10.1006/expr.1995.1049

[30] Caridha D, Hickman M, Xie L, Ngundam F, Milner E, Schenk A, et al. Updating the modified Thompson test by using whole-body bioluminescence imaging to replace traditional efficacy testing in experimental models of murine malaria. Malar J. 2019;18:38. 30767768 10.1186/s12936-019-2661-x PMC6376706

[31] Li Q, Xie L, Caridha D, Roncal N, Zeng Q, Zhang J, et al. Comparative susceptibility of different mouse strains to liver-stage infection with *Plasmodium berghei* Sporozoites assessed using in vivo imaging. Mil Med. 2017;182:360–8. 28291500 10.7205/MILMED-D-16-00090

[32] Nicklas DA, Maggioncalda EC, Story-Roller E, Eichelman B, Tabor C, Serio AW, et al. Potency of Omadacycline against *Mycobacteroides abscessus* clinical isolates in vitro and in a mouse model of pulmonary infection. Antimicrob Agents Chemother. 2022;66:e0170421. 34662184 10.1128/AAC.01704-21 PMC8765394

[33] Li Q, Gerena L, Xie L, Zhang J, Kyle D, Milhous W. Development and validation of flow cytometric measurement for parasitemia in cultures of P falciparum vitally stained with YOYO-1. Cytometry A. 2007;71:297–307. 17279569 10.1002/cyto.a.20380

[34] Xie L, Li Q, Johnson J, Zhang J, Milhous W, Kyle D. Development and validation of flow cytometric measurement for parasitaemia using autofluorescence and YOYO-1 in rodent malaria. Parasitology. 2007;134:1151–62. 17445324 10.1017/S0031182007002661

[35] Hsu HC, Li D, Zhan W, Ye J, Liu YJ, Leung A, et al. Structures revealing mechanisms of resistance and collateral sensitivity of *Plasmodium falciparum* to proteasome inhibitors. Nat Commun. 2023;14:8302. 38097652 10.1038/s41467-023-44077-2 PMC10721928

[36] Pottenger AE, Roy D, Srinivasan S, Chavas TEJ, Vlaskin V, Ho DK, et al. Liver-targeted polymeric prodrugs delivered subcutaneously improve tafenoquine therapeutic window for malaria radical cure. Sci Adv. 2024;10:4492. 10.1126/sciadv.adk4492 PMC11029812 38640243

[37] Rosado-Quiñones AM, Colón-Lorenzo EE, Pala ZR, Bosch J, Kudyba K, Kudyba H, et al. Novel hydrazone compounds with broad-spectrum antiplasmodial activity and synergistic interactions with antimalarial drugs. Antimicrob Agents Chemother. 2024;68:e0164323. 38639491 10.1128/aac.01643-23 PMC11620517

[38] Vanachayangkul P, Im-Erbsin R, Tungtaeng A, Kodchakorn C, Roth A, Adams J, et al. Safety, pharmacokinetics, and activity of high-dose ivermectin and chloroquine against the liver stage of *Plasmodium cynomolgi* Infection in Rhesus Macaques. Antimicrob Agents Chemother. 2020;64:e00741-e820. 32660993 10.1128/AAC.00741-20 PMC7449176

[39] Roth A, Adapa SR, Zhang M, Liao X, Saxena V, Goffe R, et al. Unraveling the *Plasmodium vivax* sporozoite transcriptional journey from mosquito vector to human host. Sci Rep. 2018;8:1–20. 30111801 10.1038/s41598-018-30713-1 PMC6093925

[40] Roth A, Maher SP, Conway AJ, Ubalee R, Chaumeau V, Andolina C, et al. A comprehensive model for assessment of liver stage therapies targeting *Plasmodium vivax* and *Plasmodium falciparum*. Nat Commun. 2018;9:1–16. 29743474 10.1038/s41467-018-04221-9 PMC5943321

[41] Wang Y, Jadhav A, Southal N, Huang R, Nguyen DT. A grid algorithm for high throughput fitting of dose-response curve data. Curr Chem Genomics. 2010;4:57–66. 21331310 10.2174/1875397301004010057 PMC3040458

[42] Hunter JD. Matplotlib: A 2D graphics environment. Comput Sci Eng. 2007;9:90–5.

[43] Pedregosa F, Varoquaux G, Gramfort A, Michel A, Thirion B, Grisel O, et al. Scikit-learn: machine learning in Python. JMLR. 2011;12:2825–30.

[44] Lam SK, Pitrou A, Seibert S. Numba: a LLVM-based Python JIT compiler. In Proceedings of the Second Workshop on the LLVM Compiler Infrastructure in HPC. 2015, Association for Computing Machinery: Austin, Texas. p. Article 7.

[45] Harris CR, Millman KJ, van der Walt SJ, Gommers R, Virtanen P, Cournapeau D, et al. Array programming with NumPy. Nature. 2020;585:357–62. 32939066 10.1038/s41586-020-2649-2 PMC7759461

[46] Starzengruber P, Thriemer K, Haque R, Khan WA, Fuehrer HP, Siedl A, et al. Antimalarial activity of tigecycline, a novel glycylcycline antibiotic. Antimicrob Agents Chemother. 2009;53:4040–2. 19596882 10.1128/AAC.00312-09 PMC2737841

[47] Ribatski-Silva D, Bassi CL, Martin TOG, Alves-Junior E, Gomes LT, Fontes CJF. *In vitro* antimalarial activity of tigecycline against *Plasmodium falciparum* culture-adapted reference strains and clinical isolates from the Brazilian Amazon. Rev Soc Bras Med Trop. 2014;47:110–2. 24553805 10.1590/0037-8682-0013-2012

[48] Held J, Zanger P, Issifou S, Kremsner PG, Mordmüller B. In vitro activity of tigecycline in *Plasmodium falciparum* culture adapted strains and clinical isolates from Gabon. Int J Antimicrob Agents. 2010;35:587–9. 20227854 10.1016/j.ijantimicag.2010.02.003

[49] Koehne E, Kreidenweiss A, Adegbite BR, Manego RZ, McCall MB, Mombo-Ngoma G, et al. In vitro activity of eravacycline, a novel synthetic halogenated tetracycline, against the malaria parasite *Plasmodium falciparum*. J Glob Antimicrob Resist. 2020;24:93–7. 33301999 10.1016/j.jgar.2020.11.024

[50] Newton PN, Chaulet JF, Brockman A, Chierakul W, Dondorp A, Ruangveerayuth R, et al. Pharmacokinetics of oral doxycycline during combination treatment of severe falciparum malaria. Antimicrob Agents Chemother. 2005;49:1622–5. 15793155 10.1128/AAC.49.4.1622-1625.2005 PMC1068593

[51] Sahu R, Walker LA, Tekwani BL. In vitro and in vivo anti-malarial activity of tigecycline, a glycylcycline antibiotic, in combination with chloroquine. Malar J. 2014;414:1–7. 10.1186/1475-2875-13-414 PMC4216846 25336038

[52] Jacobs RL, Koontz LC. *Plasmodium berghei*: development of resistance to clindamycin and minocycline in mice. Exp Parasitol. 1976;40:116–23. 780118 10.1016/0014-4894(76)90073-4

[53] Briolant S, Wurtz N, Zettor A, Rogier C, Pradines B. Susceptibility of *Plasmodium falciparum* isolates to doxycycline is associated with pftetQ sequence polymorphisms and pftetQ and pfmdt copy numbers. J Infect Dis. 2010;201:153–9. 19929377 10.1086/648594

[54] Achieng AO, Ingasia LA, Juma DW, Cheruiyot AC, Okudo CA, Yeda RA, et al. Reduced *in vitro* doxycycline susceptibility in *Plasmodium falciparum* field isolates from Kenya is associated with PfTetQ KYNNNN sequence polymorphism. Antimicrob Agents Chemother. 2014;58:5894–9. 25070109 10.1128/AAC.02788-13 PMC4187988

[55] Madamet M, Gaillard T, Velut G, Ficko C, Houzé P, Bylicki C, et al. Malaria prophylaxis failure with doxycycline, Central African Republic, 2014. Emerg Infect Dis. 2015;21:1485–6. 26196738 10.3201/eid2108.150524 PMC4517722

[56] Gaillard T, Madamet M, Pradines B. Tetracyclines in malaria. Malar J. 2015;14:445. 26555664 10.1186/s12936-015-0980-0 PMC4641395

[57] Zurawski DV, Serio AW, Black C, Pybus B, Akers KS, Deck DH, et al. A Review of omadacycline for potential utility in the military health system for the treatment of wound infections. Mil Med. 2024;189:e1353–61. 37963013 10.1093/milmed/usad417 PMC11110612

